# TGF-β from the Porcine Intestinal Cell Line IPEC-J2 Induced by Porcine Circovirus 2 Increases the Frequency of Treg Cells via the Activation of ERK (in CD4^+^ T Cells) and NF-κB (in IPEC-J2)

**DOI:** 10.3390/v14112466

**Published:** 2022-11-08

**Authors:** Xuewei Liu, Yang Wang, Cheng Han, Qiuming Li, Xiaolin Hou, Qinye Song, Shuanghai Zhou, Huanrong Li

**Affiliations:** 1 College of Animal Science and Technology, Beijing University of Agriculture, No. 7 Beinong Road, Changping District, Beijing 102206, China; 2 College of Veterinary Medicine, Hebei Agricultural University, Baoding 071000, China

**Keywords:** porcine circovirus 2, intestinal porcine epithelial cells, Foxp3^+^ regulatory T cells, TGF-β, ERK, NF-κB

## Abstract

Porcine circovirus 2 (PCV2) causes immunosuppression. Piglets infected with PCV2 can develop enteritis. Given that the gut is the largest immune organ, however, the response of the gut’s immune system to PCV2 is still unclear. Here, IPEC-J2 cells with different treatments were co-cultured with PBMC or CD4^+^ T cells (Transwell). Flow cytometry and Western blotting revealed that PCV2-infected IPEC-J2 increased the frequency of CD4^+^ T cells among piglets’ peripheral blood mononuclear cells (PBMCs) and caused CD4^+^ T cells to undergo a transformation into Foxp3^+^ regulatory T cells (Treg cells) via activating CD4^+^ T ERK. Cytokines production and an inhibitor assay showed that the induction of Tregs by PCV2-infected IPEC-J2 was dependent on TGF-β induced by PCV2 in IPEC-J2, which was associated with the activation of NF-κB. Taken together, PCV2-infected IPEC-J2 activated NF-κB to stimulate the synthesis of TGF-β, which enhanced the differentiation of CD4^+^ T cells into Treg cells through the activation of ERK in CD4^+^ T cells. This information sheds light on PCV2′s function in the intestinal immune system and suggests a potential immunosuppressive mechanism for PCV2 infection.

## 1. Introduction

Porcine circovirus type 2 (PCV2) is associated with postweaning multisystemic wasting syndrome (PMWS) and other syndrome diseases collectively known as porcine-circovirus-associated disease (PCVAD). PCVAD has become one of the world’s most economically significant pig diseases [1]. In PCV2-infected piglets, the number of dendritic cells (DCs), B cells, NK cells, T cells, CD4^+^ T and CD8^+^ T lymphocytes is downregulated, whereas the number of monocytes and granulocytes is upregulated [2,3,4]. Thus, PCV2 infection is thought to influence the immune system of the host, thereby increasing the chance of contracting other harmful infections [5]. Immunosuppression is the most critical characteristic of PCV2 infection.

PCV2-associated enteritis is one of the less-commonly recognized clinical manifestations of PCVAD in growing pigs. Studies have shown that PCV2 was found in the intestine of piglets infected with PCV2 [6] and was able to proliferate in the porcine intestinal cell line IPEC-J2, which was isolated from the jejunum of a neonatal unsuckled piglet [7] and could serve as a model for the study of innate immune responses [8]. Under natural and experimental conditions, PCV2 can produce enteritis independently of other enteric pathogens [9,10]. Enteritis caused by PCV2 infection was characterized by ulcerative, necrosuppurative colitis and the granulomatous inflammation of Peyer’s patches with infiltrates of epithelioid macrophages and large multinucleated cells [9,11]. Given that the gut is the largest immunologic organ and that Peyer’s patches are a critical component of the gut-associated immune system, we questioned whether the immunosuppression of PCV2 infection was affected by changes in intestinal immune function [12,13].

We, and others, have previously established that PCV2 infection increases the levels of cytokines and the frequency of immune cells in the colon, indicating that intestinal immune function may be significantly altered by PCV2 infection [14,15]. However, the mechanisms contributing to these variances are unclear.

The purpose of this study is to examine how PCV2 impacts gut immunity and its underlying mechanisms in vitro. To determine the efficacy of PCV2-infected IPEC-J2 in regulating the function of intestinal cells, we first developed a co-culture model by assessing the frequency changes of each kind of immune cell in PBMCs or CD4^+^ T cells in the presence of IPEC-J2. PCV2-infected IPEC-J2 enhanced the frequency of CD4^+^ T cells in PBMCs and prompted CD4^+^ T cells to convert into Treg cells, according to the findings. Then, we investigated the factors between CD4^+^ T cells and IPEC-J2 that lead to the expansion of Treg cells. This study gives theoretical evidence for PCV2 immunosuppression and a greater understanding of how PCV2 infection regulates gut immune function.

## 2. Materials and Methods

### 2.1. Animal, Cell and Virus

Three thirty-day-old specific-pathogen-free (SPF) large white piglets (free of PCV1, PCV2, porcine respiratory and reproductive syndrome virus, classical swine fever virus, pseudorabies virus and mycoplasma hyopneumoniae) were purchased from the Beijing Centre of SPF Swine Breeding and Management. Intestinal porcine epithelial cells J2 (IPEC-J2) (from DSMZ, NO 701) were cultured in DMEM (Gibco, Grand Island, NY, USA) supplemented with 10% FBS (BI, Kibbutz Beit, Israel) and incubated in an atmosphere of 5% CO_2_ at 37 °C (Thermo Fisher Scientific, Waltham, MA, USA). The PCV2 strain SD/2008 (GenBank accession number: GQ174519) was isolated and identified by the Animal Infectious Disease Laboratory at Hebei Agricultural University.

### 2.2. Isolation of Peripheral Blood Mononuclear Cells (PBMCs), Total T Cells and CD4^+^ T Cells

The SPF piglets’ PBMCs were isolated by Lymphoprep (Nycomed Pharma AS, Oslo, Norway) sedimentation and cultured in RPMI 1640 medium (BioWhittaker, Verviers, Belgium) supplemented with 10 mM HEPES (pH 7.5; Sigma-Aldrich, Deisenhofen, Germany), 2 mM L-glutamine and 100 U/mL penicillin (Sigma). The CD3^+^ T cells were then separated using CD3 MicroBeads (Miltenyi, Bergisch Gladbach, Germany) with positive selection (purity >95%). The T cells were activated with anti-CD3/CD28 mAbs (the results are shown in Figure S1) and then the CD4+ T cells were isolated using CD4 MicroBeads (Miltenyi, Bergisch Gladbach, Germany) with positive selection (purity >95%).

### 2.3. Co-Culture System

The IPEC-J2 cells (2 × 10^6^ cells/well) were seeded, and formed a monolayer on the bottom chambers of the transwell system (Corning, NY, USA). Then, PCV2 (MOI = 1) was infected with IPEC-J2. In the upper chambers, fresh PBMCs, total T cells or CD4^+^ T cells (3 × 10^6^ cells/well) were added after 48 h. On the third day of co-culture, the top chambers were analyzed by flow cytometry for the presence of cells. All cells were grown at 37 °C in a 5% CO_2_ atmosphere in DMEM media (Gibco, Grand Island, NY, USA) supplemented with 10% FBS. 

### 2.4. Inhibitor Assay 

Before co-culture, the CD4^+^ T cells were pre-treated with DMSO (the vehicle) or AZD8330 (ERK inhibitor; MedChemExpress, NJ, USA) for 24 h. The IPEC-J2 cells were treated with either DMSO (vehicle) or PG490 (NF-κB inhibitor; MedChemExpress, NJ, USA). The IPEC-J2 cells were infected with PCV2 for 48 h after 12 h, and were then co-cultured with CD4^+^ T cells.

### 2.5. Extraction of Total RNA and Quantitative PCR

Total RNA was extracted from cells using a Total RNA Kit I (Omega Bio-tek, Norcross, GA, USA). The RNA was then reverse transcribed using a HiScript II 1st Strand cDNA Synthesis Kit (Vazyme, Nanjing, China) following the manufacturer’s instructions. Quantitative RT-PCR was performed using AceQ^®^ qPCR SYBR^®^ Green Master Mix (Vazyme, Nanjing, China). Data were presented as fold change in gene expression normalized to β-actin and relative to the mock infected control. Each reaction was performed in triplicate, and the data were calculated as means (M) ± SEM. The primer sequences for the genes are shown in Table 1.

### 2.6. Confocal Microscopy

The CD4^+^ T cells were fixed with 4% paraformaldehyde in PBS at 4 °C for 30 min. After three washes with ice-cold PBS, these cells were permeabilized with 0.1% Triton X-100 for 15 min and blocked in 5% bovine serum albumin (BSA) in PBS for 1 h at 37 °C. Then, they were incubated with appropriate primary antibodies (rabbit anti-p-ERK1/2 1:1000; Abcam, Cambridge, MA, USA) for 1 h at 37 °C, washed with PBS, and incubated with Alexa Fluor 594-conjugated goat anti-rabbit IgG (H-L) (1:200; Beyotime, Shanghai, China) for 1 h at 37 °C in the dark. The nuclei were stained with DAPI (Invitrogen, Carlsbad, CA, USA) for 15 min at room temperature. The stained cells were imaged and analyzed using the Axiovision automatic measurement application with a Nikon A1 confocal microscope (Nikon A1; Nikon, Tokyo, Japan).

### 2.7. Flow Cytometry

For the flow cytometric characterization of different lymphocytes, the cells were stained with directly conjugated monoclonal antibodies specific for CD3, IL-17A (BD Pharmingen, San Diego, CA, USA), CD21, CD4, CD48, SWC3a (Southern Biotech, Birmingham, AL, USA), CD1a, IFN-γ (Abcam, Cambridge, MA, USA), SLA II (BIO-RAD, Hercules, CA, USA), IL-4 (R&D systems, Minneapolis, MN), IL-10 (Invitrogen, Carlsbad, CA, USA), CD152 (Ancell, Bayport, MN, USA) and Foxp3 (eBioscience, San Diego, CA, USA), or with appropriate isotype controls. The flow cytometric analysis was performed using an Attune NxT flow cytometer (Invitrogen, Carlsbad, CA, USA) with FlowJo software (Tree Star Inc., MA, USA).

### 2.8. Western Blot Assay

The cells were lysed in lysis buffer (Beyotime, Shanghai, China) for 15 min on ice, then separated by sodium dodecyl sulfate-polyacrylamide gel electrophoresis (SDS-PAGE) and transferred to a nitrocellulose membrane. After blocking the membrane with 5% low-fat milk for 2 h at room temperature, the membrane was probed with the appropriate primary antibodies for 2 h at room temperature. HRP-conjugated goat anti-mouse and -rabbit IgG (H-L) secondary antibodies were treated with membranes (1:1000; Beyotime, Shanghai, China). The Tanon 5200 chemiluminescence imaging system was utilized to detect bound proteins (Tanon, Shanghai, China). The bands were measured using ImageJ (Version 5.1) and normalized to β-actin levels for quantitative analysis. The primary antibodies used in Western blotting (WB) (anti-β--actin, anti-ERK1/2, anti-phospho-ERK1/2, anti-PI3K, anti-phospho-PI3K, anti-AKT and anti-phospho-AKT) were purchased from Abcam.

### 2.9. ELISA of TGF-β

The concentrations of TGF-β in cell culture supernatants were measured using sandwich ELISA kits (R&D systems, Minneapolis, MN, USA) according to the manufacturer’s protocols.

### 2.10. Ethics Statement

All animal experiments conformed to the rules of the National Guidelines for Housing and Care of Laboratory Animals (China) and were performed after obtaining the approval of the Institutional Animal Care and Ethics Committee of Beijing University of Agriculture (approval No. SYXK2019-0005). All piglets were housed in the animal facility of Beijing University of Agriculture (Beijing, China).

### 2.11. Statistical Analysis

All statistical analyses were performed using GraphPad Prism 7.0 software (Version X; La Jolla, CA, USA). The results were expressed as the mean ± SD. The significances of the differences among groups were determined by one-way or two-way analysis of variance. Differences with *p*-values < 0.05 were considered significant and designated with an asterisk (*) or pound sign (#) in the figures. Unless indicated otherwise, the experiments were performed in triplicate (*n* = 3).

## 3. Results

### 3.1. PCV2-Infected IPEC-J2 Leads to the Expansion of the Treg Cells Population

**Figure 1:**
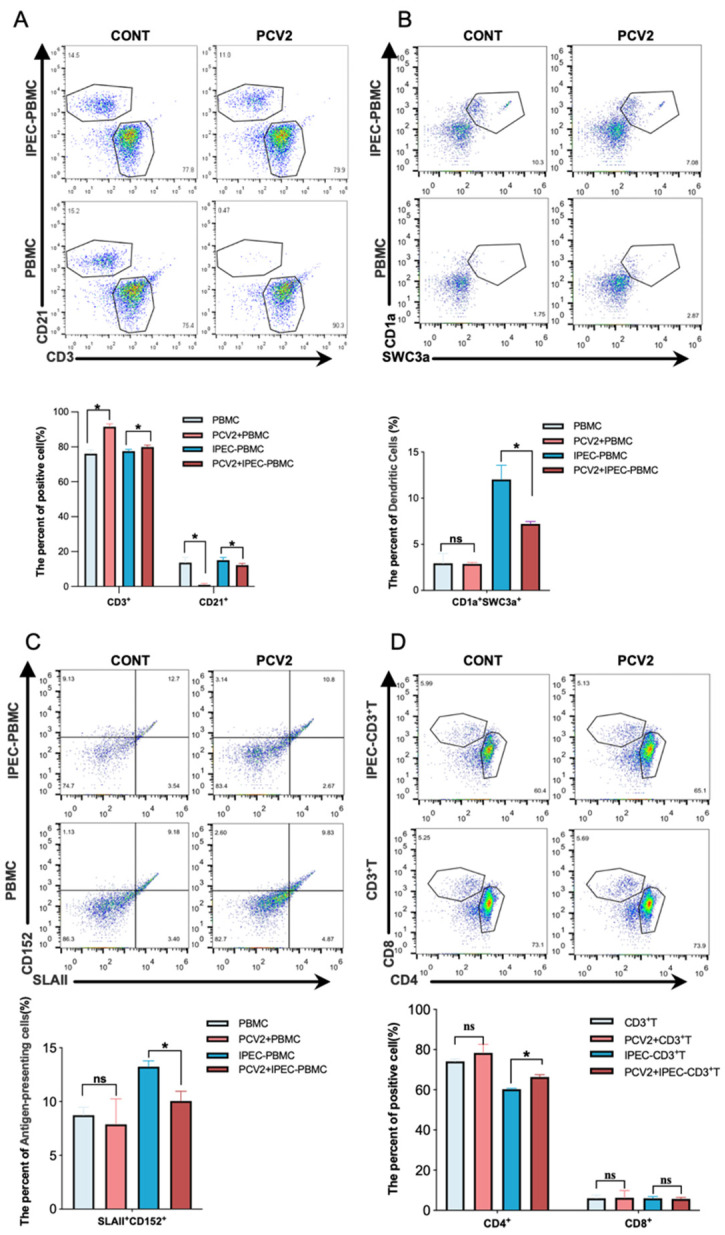
**The changes in the frequency of immune cells caused by PCV2-infected IPEC-J2.** On the bottom chambers of the transwell system, IPEC-J2 cells were seeded and then infected with PCV2 (MOI = 1) for 48 h. In the upper chambers, fresh PBMCs were added. Flow cytometry was used to determine the frequencies of total T cells, B cells (**A**), DCs (**B**) and APCs (**C**) in the upper chambers after 3 days of co-culture. PBMCs were also individually cultured or infected with PCV2 directly for 48 h as a control. Using CD3 MicroBeads, total T cells were collected and co-cultured with IPEC-J2 or IPEC-J2 infected with PCV2. After 3 days of co-culture, the frequencies of CD4^+^ T cells and CD8^+^ T cells were determined by flow cytometry (**D**). All assays were performed in triplicate, with three technical repeats for each sample. * *p* < 0.05.

To investigate whether PCV2-infected IPEC-J2 can alter intestinal immunity, we created an in vitro model of intestinal epithelial cells and intestinal immune cells utilizing a co-culture system of IPEC-J2 (infected with PCV2 or not infected with PCV2) and PBMC. PCV2-infected IPEC-J2 decreased the frequencies of B cells (CD21^+^) (Figure 1A), dendritic cells (CD1a^+^SWC3a^+^) (Figure 1B) and antigen-presenting cells (CD152^+^SLA II^+^) (Figure 1C) in PBMCs while increasing the frequency of T cells (CD3^+^) (Figure 1D). To remove the effect of PCV2 on PBMCs, we isolated a group of PCV2-infected PBMCs and found no variation in the frequency of immune cells. To illustrate the effect of PCV2-infected IPEC-J2 on T cells, total T cells were isolated using CD3 MicroBeads and subsequently co-cultured with IPEC-J2- or PCV2-infected IPEC-J2. Using flow cytometry, the frequencies of CD4^+^ T and CD8^+^ T cells were determined. As shown in Figure 1D, there was no difference in the frequency of CD8^+^ T cells among CD3^+^ T cells; however, we observed an increase in the frequency of CD4^+^ T cells in the PCV2-infected IPEC-J2 group compared with the IPEC-J2 group, suggesting that PCV2-infected IPEC-J2 could upregulate the content of CD4^+^ T cells, but not CD8^+^ T cells.

**Figure 2:**
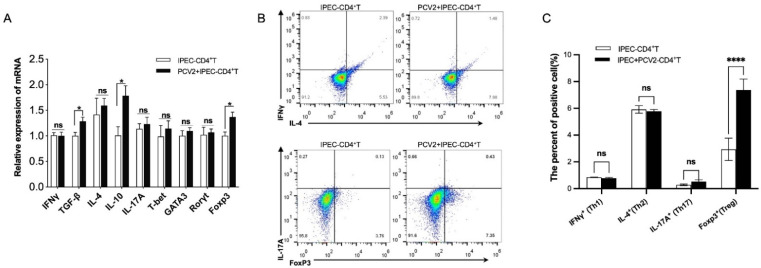
**The changes in the frequencies of different CD4^+^ T cells caused by PCV2-infected IPEC-J2.** CD4^+^ T cells were isolated using CD4 MicroBeads; the purify was >95%. IPEC-J2 cells were seeded and infected with PCV2 or not infected for 48 h, and were then co-cultured with CD4^+^ T cells for 3 days. The mRNA expression of IFN-γ, TGF-β, IL-4, IL-10, IL-17A, T-bet, GATA3, Rorγt and Foxp3 in CD4^+^ T cells was detected by qPCR (**A**). Flow cytometry was used to detect the percentages of Th1, Th2, Th17 and Treg cells in the CD4^+^ T cells (**B**). Graph represents the frequencies of Th1, Th2, Th17 and Treg cells in PCV2-infected or not infected groups (**C**). All assays were performed in triplicate, with three technical repeats for each sample. **** *p* < 0.0001; * *p* < 0.05; ns, No Significant.

Given that CD4^+^ T cells differentiate into numerous subgroups of effector T helper cells and regulatory T cells (Treg cells), they have been described as reflecting the phenotypes of Th cells based on the expression of various transcription factors, including T-bet (Th1), GATA3 (Th2), Rorγt (Th17) and Foxp3 (Treg cells), and altering the expressions of cytokines, such as IFN (Th1), IL-4 (Th2), IL-17A (Th17), TGF-β and IL-10 (Treg cells) [16,17,18]. We co-cultured CD4^+^ T cells with IPEC-J2 to determine the effect of PCV2-infected IPEC-J2 on the subset of CD4^+^ T cells. qPCR was performed to find the expressions of the cytokines and transcription factors described above. The mRNA expressions of IFN-γ, IL-4, IL-17A, T-bet, GATA3 and Rorγt were not significantly different in the co-culture group of PCV2-infected IPEC-J2 and CD4^+^ T cells (PCV2-IPEC+CD4^+^ T group) compared to the IPEC+CD4^+^ T group, but TGF-β, IL-10 and Foxp3 were significantly upregulated (*p* < 0.05) (Figure 2A). Flow cytometry supported these findings as well. While the frequency of Foxp3^+^ cells increased significantly in the PCV2-IPEC+CD4^+^ T group (*p* < 0.05), the frequencies of IFNγ^+^ cells, IL-10^+^ cells and IL-17A^+^ cells remained unchanged (Figure 2B). PCV2-infected IPEC-J2 impacted the amount of Treg cells, as evidenced by these results.

### 3.2. PCV2-Infected IPEC-J2 Enhanced the Frequency of Treg Cells by ERK Activation in CD4^+^ T

**Figure 3:**
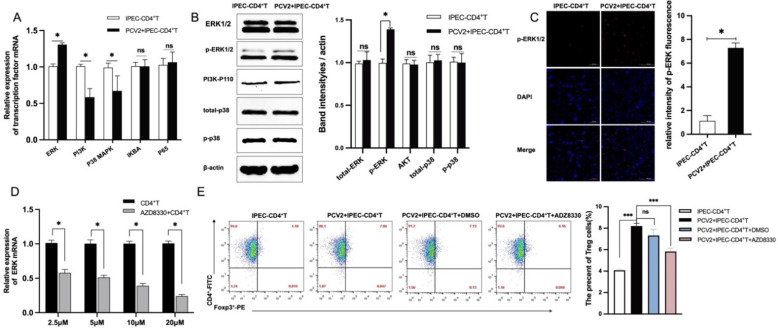
**PCV2-infected IPEC-J2 increased the amount of Treg by activating ERK in CD4^+^ T cells.** IPEC-J2 were infected with PCV2 or not infected for 48 h, and were then co-cultured with CD4^+^ T cells for 3 days. The mRNA expressions of ERK, PI3K, P38, IKBA and NF-κB P65 in CD4^+^ T cells were detected by qPCR (**A**). Western blotting was used to test the protein expressions of p110 (PI3K- p110), ERK1/2 and phosphorylated ERK1/2 (p-ERK1/2), total p38 and phosphorylated p38(p-p38). The expression of each protein was quantified using ImageJ and normalized to β-actin levels (**B**). A confocal laser was used to detect the p-ERK1/2 in CD4^+^ T cells after 6 h co-culture, and the intensity of p-ERK1/2 fluorescence was quantified by ImageJ (**C**). qPCR was used to detect the mRNA expressions of ERK in CD4^+^ T cells, which were treated with AZD8330 (AZD8330+ CD4^+^ T) or DMSO (CD4^+^ T) for 1 h (**D**). IPEC-J2 cells were infected with PCV2 or not infected for 48 h, and were then co-cultured with CD4^+^ T cells (treatment with 20 μM AZD8330/DMSO for 1 h in advance) for 3 days. Flow cytometry was used to detect the percentage of Treg cells (**E**). All assays were performed in triplicate, with three technical repeats for each sample. *** *p* < 0.001; * *p* < 0.05; ns, No Significant.

CD4^+^ T cell differentiation is mediated by ERK, PI3K, P38, IKBA and NF-κB [19,20,21,22]. To outline the contributions of these signal pathways to the increase of Treg cells by PCV2-infected IPEC-J2, qPCR was performed firstly to quantify the mRNA expression of ERK, PI3K, P38, IKBA and NF-κB P65 in CD4^+^ T cells. As shown in Figure 3A, PCV2-infected IPEC-J2 altered the mRNA levels of ERK, PI3K and P38 in CD4^+^ T cells. We further confirmed the protein expression of p110 (the subunit of PI3K), ERK1/2 and phosphorylated ERK1/2 (pERK1/2), total p38 and phosphorylated p38(p-p38) by WB. pERK1/2 were considerably elevated by stimulations of the PCV2-infected IPEC-J2 after 6 h co-culture. IPEC-J2 did not activate ERK on its own. However, no significant variations in p110, p38 or p-p38 were found (Figure 3B). We also used confocal microscopy to look for phosphorylated ERK in the CD4^+^ T cells. The results demonstrated that PCV2-infected IPEC-J2 raised the expression of phosphorylated ERK in the CD4^+^ T cells more than IPEC-J2 alone (Figure 3C). This data suggests that PCV2-infected IPEC-J2 activate ERK in CD4^+^ T cells.

To determine whether the expansion of Treg cells is dependent on ERK activation, CD4^+^ T cells were treated with AZD8330 (a selective ERK inhibitor). The best AZD8330 concentration was screened firstly. AZD8330 suppressed ERK expression in CD4^+^ T cells, as demonstrated in Figure 3D, and 20 μM of AZD8330 worked well. The CD4^+^ T cells were then pre-treated with 20 μM AZD8330 or DMSO (the vehicle) and co-cultured with IPEC-J2 (infected or not infected). In the PCV2+IPEC-CD4^+^ T group, the frequency of Treg cells increased after 3 days of co-culture, which was consistent with the previous result. There were no significant differences between the PCV2+IPEC-CD4^+^ T and DMSO-treated groups. Treatment with an ERK inhibitor (AZD8330) resulted in a significantly lower frequency of Treg cells as compared with the PCV2-IPEC+CD4^+^ T group (Figure 3E). These findings emphasize that co-culture with PCV2-infected IPEC-J2 raises ERK activation in CD4^+^ T cells and increases the frequency of Treg cells.

### 3.3. The Increase of Treg Cells Was Dependent on TGF-β from the PCV2-Infected IPEC-J2 and the NF-κB Activation in IPEC-J2

**Figure 4:**
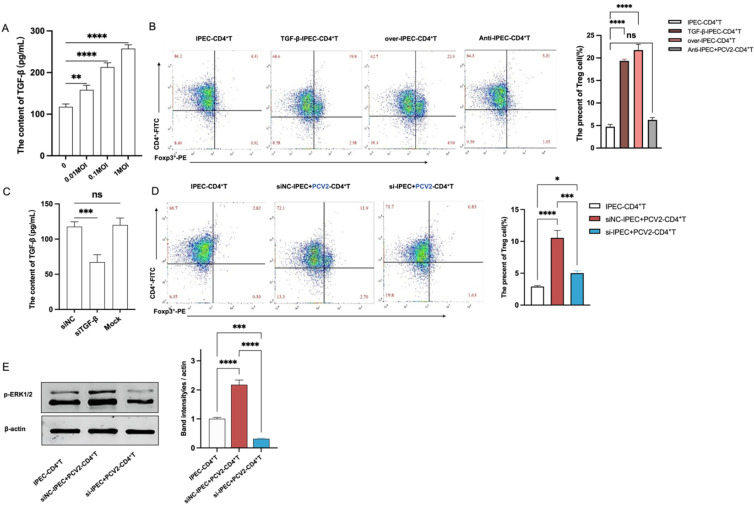
**PCV2-infected IPEC-J2 increased the amount of Tregs in response to TGF-β from IPEC-J2.** The production of TGF-β from PCV2-infected IPEC-J2 was detected by ELISA (**A**). Four groups were created: the IPEC-CD4^+^ T cell group, the TGF-β-IPEC+CD4^+^ T cell group (adding 230 pg/mL TGF-β to the IPEC-J2 culture medium), the over-IPEC+CD4^+^ T cell group (transfected 1μg of plasmid expressed TGF-β in IPEC-J2 24 h in advance) and the anti-IPEC-PCV2+CD4^+^ T cell group (adding 2 μg/mL antibody against TGF-β to the IPEC culture media). After 3 days co-culture, flow cytometry was used to determine the frequencies of Treg cells (**B**). IPEC-J2 was transfected with 100 nM siTGF-β or siNC for 24 h, and the production of TGF-β from IPEC-J2 was detected by ELISA (**C**). The IPEC-J2 transfected with siTGF-β or siNC were infected with PCV2 and then co-cultured with CD4^+^ T cell for 3 days. The percentages of Treg cells were tested by flow cytometry (**D**). The expression of p-ERK1/2 in CD4^+^ T cell from (**D**) was determined by Western blotting, quantified using ImageJ and normalized to β-actin levels (**E**). All assays were performed in triplicate, with three technical repeats for each sample. **** *p* < 0.0001; *** *p* < 0.001; ** *p* < 0.01; * *p* < 0.05; ns, No Significant.

Studies have reported that CD4^+^ T cells can differentiate into Treg cells in the presence of cytokines such as TGF-β or IL-10 or through interactions between cells [23,24]. PCV2 infection has been demonstrated to promote TGF-β production in vitro and in vivo by previous studies, including our own [25,26]. As depicted in Figure 4A, the production of TGF-β increased as the PCV2 dose increased. We thus assume that TGF-β was necessary for the differentiation of CD4^+^ T cells into Treg cells in PCV2-infected IPEC-J2. To aid in the identification our presumption, the IPEC-J2 cells with different treatments were co-cultured with CD4^+^ T cells, and the groups were the IPEC-CD4^+^ T cell group, the TGF-β-IPEC+CD4^+^ T cell group (adding TGF-β to the IPEC-J2 culture medium), the over-IPEC+CD4^+^ T cell group (overexpressing TGF-β in IPEC-J2) and the anti-IPEC-PCV2+CD4^+^ T cell group (adding an antibody against TGF-β to the IPEC-J2 culture media; the TGF-β protein levels after antibody treatment in IPEC-J2 are shown in Figure S2.), respectively. Flow cytometry was used to determine the frequency of Treg cells after 6 h of co-culture. As shown in Figure 4B, addition of TGF-β in the culture medium of IPEC-J2 and the over-expression of TGF-β in IPEC-J2 increased the frequency of Treg cells, whereas the application of anti-TGF-β antibody in the IPEC-J2 culture medium resulted in the virtual disappearance of the role of PCV2-infected IPEC-J2, indicating that TGF-β does have a stimulatory effect on the amount of Treg cells in the co-culture system. In the co-culture system, our data demonstrates that TGF-β plays a crucial role in the differentiation of CD4^+^ T cells into Treg cells. To examine the effect of PCV2-induced TGF-β on CD4^+^ T cells, a small interfering RNA (siRNA) that specifically targets TGF-β (siTGF-β) was created, and IPEC-J2 were transfected with siTGF-β, i.e., si-IPEC. As shown in Figure 4C, the production of TGF-β was decreased in the IPEC-J2 which transfected with siTGF-β but not the IPEC-J2 which transfected with siNC (the nontargeting control siRNA). Later, the cells were co-cultured with CD4^+^ T cells and infected with PCV2 after 24 h. IPEC-J2 infected with PCV2 also increased the amount of Treg cells after three days of co-culture. In contrast, the frequency of Treg cells in the si-IPEC-PCV2+CD4^+^ T cell group decreased significantly, but did not reach the level observed in the PCV2-uninfected group (Figure 4D). The results revealed that TGF-β from IPEC-J2 induced by PCV2 was responsible for the increase in Treg cell differentiation. Given that PCV2-infected IPEC-J2 produces an increase in Treg cells by activating ERK in CD4^+^ T cells, we investigated whether inhibiting TGF-β inside IPEC-J2 impacts ERK activation in CD4^+^ T cells. As expected, the degree of ERK activation differed between the si-IPEC-PCV2+CD4^+^ T cell group and the PCV2-CD4^+^ T cell group: siTGF-β application resulted in a significantly reduced level of ERK activation (Figure 4E).

**Figure 5:**
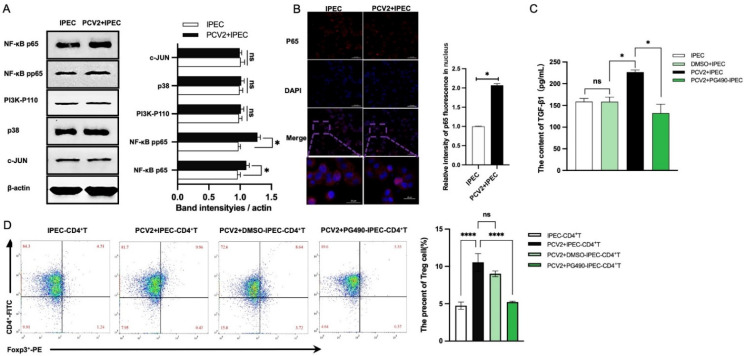
The increase in the number of Tregs caused by PCV2-infected IPEC-J2 is dependent on the activation of NF-κB in IPEC-J2. Western blotting was used to test the protein expressions of p110 (PI3K- p110), total p38, c-JUN, NF-κB p65 and the phosphorylated NF-κB p65 (NF-κB pp65). The expression of each protein was quantified using ImageJ and normalized to β-actin levels (**A**). A confocal laser was used to detect the p65 in the nucleus of a CD4^+^ T cell and quantified by ImageJ (**B**). The production of TGF-β from IPEC-J2, IPEC-J2 treated with DMSO, PCV2-infected IPEC-J2 and IPEC-J2 treated with 20 μM PG490 (inhibitor of NF-κB) was detected by ELISA (C). The IPEC-J2 from (**C**) was co-cultured with a CD4^+^ T cell, and the percentage of Treg cells was tested by flow cytometry after 3 days (**D**). All assays were performed in triplicate, with three technical repeats for each sample. **** *p* < 0.0001; * *p* < 0.05; ns, No Significant.

TGF-β gene transcription can be controlled by PI3K [27,28], c-JUN [29], NF-κB [30,31] and p38 [30]. Here, we sought to determine whether PI3K, c-JUN, NF-κB and p38 were involved in TGF-β transcription in IPEC-J2 following PCV2 infection. Initially, Western blotting was used to examine the effect of PCV2 on the expressions of these proteins. Figure 5A demonstrates that the expression of phosphorylated p65 (a subunit of NF-κB) was considerably increased in PCV2-infected IPEC-J2 compared with uninfected IPEC-J2, although there were no significant changes in the expressions of other proteins. Thus, we found the nuclear staining level of p65, a standard indicator of NF-κB activation. PCV2 enhanced the nuclear translocation of p65 in IPEC-J2, indicating its positive effect on NF-κB activation in IPEC-J2 (Figure 5B). The IPEC-J2 was treated with 20 μM of PG490 (an NF-κB inhibitor) to determine whether PCV2 promoted TGF-β via the activation of NF-κB. As demonstrated in Figure 5C, treatment with PG490 dramatically decreased TGF-β expression in comparison with the control group. Hence, NF-κB played a crucial role in the PCV2-induced TGF-β expression in IPEC-J2.

Next, we analyzed the effect of NF-κB in IPEC-J2 on CD4^+^ T cells. IPEC-J2 were treated with NF-κB inhibitor (PG490) for 12 h prior to infection with PCV2 and co-cultured with CD4^+^ T cells. After 3 days, the frequency of Treg cells was considerably reduced in IPEC-J2 treated with an NF-κB inhibitor compared with the PCV2-IPEC+CD4^+^ group and the DMSO group (the vehicle) (Figure 5D).

**Figure 6:**
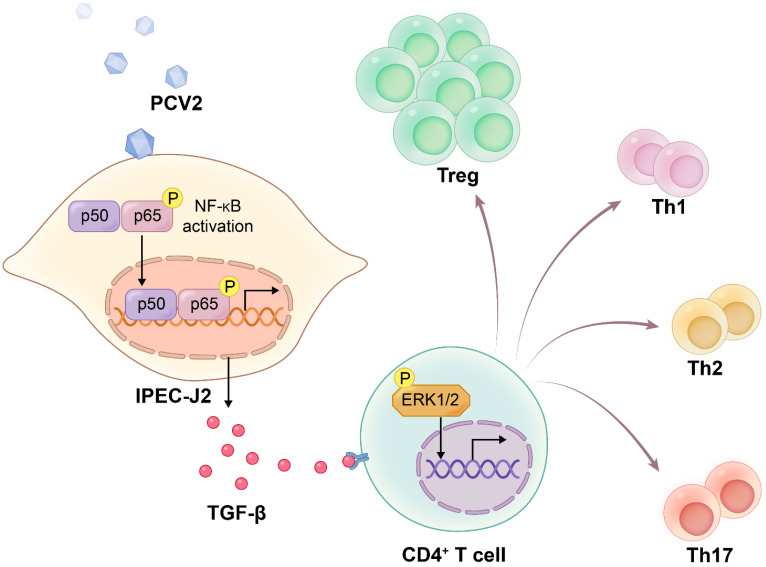
**Scheme of PCV2-infected IPEC-J2 increasing the amount of Tregs.** The infection of PCV2 on IPEC-J2 activated NF-κB, which in turn stimulated the production of TGF-β. The activation of ERK in CD4^+^ T cells was required for TGF-β to be able to promote the differentiation of CD4^+^ T cells into Treg cells.

In conclusion, the PCV2 infection of IPEC-J2 activated NF-κB to stimulate the synthesis of TGF-β. Then, TGF-β enhanced the differentiation of CD4^+^ T cells into Treg cells, dependent on the activation of ERK in CD4^+^ T cells (Figure 6).

## 4. Discussion

Swine have a full set of innate and adaptive immune effectors, making them an ideal animal model for researching the immunopathological mechanisms underlying a variety of infectious illnesses [32]. Previous research has demonstrated that PCV2 can weaken the host’s immune system, allowing additional pathogens to infect pigs [33]. The intestine is the largest immune system compartment, and is increasingly implicated in controlling the development of disease elsewhere in the body [34,35]. Virus-infected epithelial cells are capable of secreting a number of immune-related substances to regulate the immunological function of the organism. As a barrier to intestinal immunity, intestinal epithelial cells serve a crucial function in preventing the invasion of exogenous pathogens [36]. In this study, we investigated in vitro whether the PCV2 infection of the porcine intestinal cell inhibits the immunological response of the intestine. The key finding is that PCV2-infected IPEC-J2 increased the amount of Treg cells, and its mechanisms were identified in both CD4^+^ T cells and IPEC-J2.

PBMCs are a group of complicated cells primarily constituted of 80% T and B lymphocytes, 10% NK cells and 10% monocytes [37]. Due to their complicated compositions, variations in the contents of individual cells can reflect changes in the immunological function of the body [38]. In our work, a co-culture system of IPEC-J2 and PBMCs was initially developed, and then the alterations of various immune cells in PBMCs were detected in both PCV2-infected and uninfected individuals. The results demonstrate that PCV2-infected IPEC-J2 enhance the frequency of CD4^+^ T cells, but not PCV2 or IPEC-J2 alone. CD4^+^ T cells are critical mediators of immunological homeostasis, and influence immune responses by creating various effector T helper subsets, such as Th1, Th2, Th17 and regulatory T cells, in response to environmental cues such as particular stimulation [20]. Their mutual regulation and effect are essential for the body’s antiviral immunological function [16]. Following co-culture of pure CD4^+^ T cells and IPEC-J2 infected with PCV2, the number of Foxp3^+^ regulatory T cells (Treg cells) was considerably increased. Humans with persistent viral infections have been associated with increased numbers of Treg cells [39]. Treg cells serve many functions in preventing the restoration of immunological homeostasis, including the generation of anti-inflammatory cytokines and the regulation of effector T cell metabolism, as demonstrated by numerous studies [40,41]. Increased Treg cells hinder the normal antiviral response and result in immunological dysfunction [42]. Previous research demonstrated that PCV2-infected DCs dramatically boosted Treg cells [25]. Consequently, our findings also reveal a potential mechanism for immunosuppression and prolonged PCV2 infection.

Various signaling pathways might be used to excite CD4^+^ T cells, which would then develop into distinct subsets of T helper cells or regulatory T cells. By blocking the phosphorylation of the phosphoinositide 3-kinase (PI3K)/protein kinase B (AKT) signaling pathway, apremilast could regulate Treg cells [43]. Through the ERK and P38 signaling pathways, madecassic acid alters the ratio of Treg to Th17 cells [44,45]. Our research demonstrates that PCV2-infected IPEC-J2 can increase ERK activation in CD4^+^ T cells. When ERK was suppressed, Treg cell modifications disappear. The detailed upstream and downstream cell responses need additional research.

Our observations demonstrate that PCV2-infected IPEC-J2 regulated the frequency of Treg cells that were not affected by either PCV2 alone or IPEC-J2 alone, suggesting that PCV-induced IPEC-J2 secretion was responsible for the observed changes. T cells can be stimulated in the presence of TGF-β and IL-2 to produce Treg cells [46,47]. According to previous studies, PCV2 can stimulate TGF-β both in vivo and in vitro [14,48]. PCV2 infection induced an increase in TGF-β secretion in IPEC-J2 and TGF-β played a crucial role in the generation of Treg cells in our co-culture system. Using small interfering RNA (siRNA) that specifically target TGF-β (siTGF-β) decreased the number of Treg cells and decreased TGF-β expression in IPEC-J2. These findings identify TGF-β as a critical component of IPEC-J2 that was triggered by PCV2 and was responsible for the development of Treg cells. Of course, it is worth exploring whether there are other factors of PCV2 infection of IPEC-J2 that affect CD4^+^ T’s differentiation into Treg cells.

Several viruses have evolved the ability to influence the TGF-β pathway. Virus particles or viral proteins can activate TGF-β and stimulate its production in several host cells. Recent research indicates that HCV NS4 stimulates TGF-β synthesis in monocytes [49]. In vitro, the HBV infection of Kupffer cells stimulates TGF-β expression [50]. Diverse signaling pathways impact the production of TGF-β under different stimuli [27,28,29,30,31]. In our investigation, we determined that the activation of NF-κB by PCV2 was the precise cause of the increase in TGF-β in IPEC-J2. As such, it is necessary to determine the viral protein of PCV2 affecting TGF-β secretion in the future.

Our findings provide conclusive evidence that PCV2 infection activates NF-κB and causes IPEC-J2 to release TGF-β, which enhances the frequency of Treg cells through phosphorylating ERK. These results may contribute to a better understanding of how the intestinal immune system responds to PCV2 infection and the immunological pathways responsible for immunosuppression in PCV2-infected animals.

## Figures and Tables

**Table 1:** Specific primers for fluorescence quantitative PCR.

Genes	Primer Sequence (5′-3′)
IFNγ	Forward:GAGCCAAATTGTCTCCTTCTACT
IL-4	Reverse: CTGACTTCTCTTCCGCTTTCT
	Forward: GCCGGGCCTCGACTGT
IL-6	Reverse: TCCGCTCAGGAGGCTCTTC
	Forward:CTGGCAGAAAACAACTGAACC
IL-10	Reverse: TGATTCTCATCAAGCAGGTCTCC
	Forward: CGGCGCTGTCATCAATTTCTG
IL-17A	Reverse: CCCCTCTCTTGGAGCTTGCTA
	Forward: CTCTCGTGAAGGCGGGAATC
T-bet	Reverse: GTAATCTGAGGGCCGTCTGG
	Forward: ACAAACCCGATATGGCTGAGA
GATA3	Reverse: CCTGCTTGCTTCTCCTGTTC
	Forward:GCTCTACACAAAATGAAGGAC
Rorγt	Reverse: TCGTTGTGGTTTGACAGTTTGC
	Forward: TTCAGTACGTGGTGGAGTTC
Foxp3	Reverse: TGTGGTTGTCAGCGTTGTAG
	Forward: TGCCATTCGCCACAACTT
β-actin	Reverse: CCTGTCCATCCTTCTTTCCTT

## Data Availability

Not applicable.

## Supplementary Materials

The following supporting information can be downloaded at: https://www.mdpi.com/article/10.3390/v14112466/s1, Figure S1: The concentration of antibodies that activate CD4^+^ T cells; Figure S2: TGF-β protein levels after antibody treatment in IPEC-J2.
