# Supplementary material for: TGF-β from the Porcine Intestinal Cell Line IPEC-J2 Induced by Porcine Circovirus 2 Increases the Frequency of Treg Cells via the Activation of ERK (in CD4+ T Cells) and NF-κB (in IPEC-J2)
Source: Viruses. 2022 Nov 8;14(11):2466. doi: 10.3390/v14112466 (PMC9698303; doi:10.3390/v14112466)
Supplement: Supplementary file 1 [file viruses-14-02466-s001.zip › viruses-1991502-supplementary.pdf]

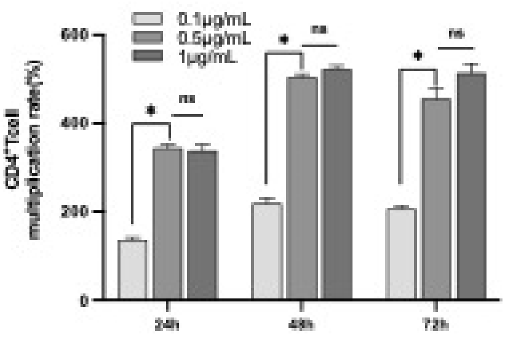

Figure S1. The concentration of antibodies that activate CD4$^+$T cells. CD4$^+$T cells were transfected with different doses of CD3/CD28. The CD25 mRNA was detected after 24 hours, 48 hours, 72 hours. All assays were performed in triplicate, with three technical repeats for each sample. **, P < 0.01; *, P < 0.05.

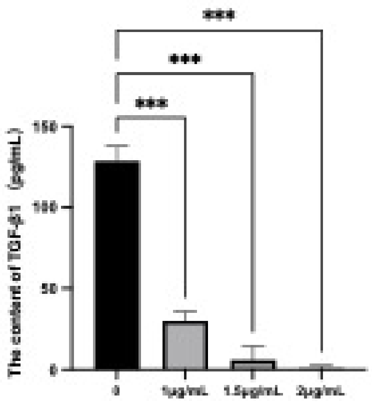

Figure S2. TGF-β protein levels after antibody treatment in IPEC-J2. TGF-β was detected with ELISA while adding the antibody to PCV2-infected IPEC-J2 after 48 hours. All assays were performed in triplicate, with three technical repeats for each sample. **, P < 0.01; *, P < 0.05.
